# Device-Removal, Reinfection, and Mortality After *Staphylococcus aureus* Bacteremia in Patients With Cardiac Implantable Electronic Devices

**DOI:** 10.1016/j.jacadv.2025.102480

**Published:** 2026-01-20

**Authors:** Kasper Høtoft Bengtsen, Melanie Vuong Le, Ketil Haugan, Berit Thornvig Philbert, Jens Brock Johansen, Christian Torp-Pedersen, Sam Riahi, Jens Cosedis Nielsen, Charlotte Larroudé, Amna Alhakak, Henning Bundgaard, Andreas Petersen, Anders Rhod Larsen, Lauge Østergaard, Emil Fosbøl, Niels Eske Bruun, Anne-Christine Ruwald

**Affiliations:** a Department of Cardiology, Zealand University Hospital, Roskilde, Denmark; b Department of Cardiology, Rigshospitalet, Copenhagen, Denmark; c Department of Cardiology, Odense University Hospital, Odense, Denmark; d Steno Diabetes Center Copenhagen, Copenhagen, Denmark; e Department of Public Health, Faculty of Health and Medical Sciences, University of Copenhagen, Copenhagen, Denmark; f Department of Cardiology, Aalborg University Hospital, Aalborg, Denmark; g Department of Cardiology, Aarhus University Hospital, Aarhus, Denmark; h Department of Cardiology, Herlev and Gentofte University Hospital, Copenhagen, Denmark; i National Reference Laboratory for Antimicrobial Resistance, Department of Bacteria, Fungi and Parasites, Statens Serum Institut, Copenhagen, Denmark; j Department of Clinical Medicine, Faculty of Health and Medical Sciences, University of Copenhagen, Copenhagen, Denmark; k Institute of Clinical Medicine, University of Aalborg, Aalborg, Denmark

**Keywords:** all-cause mortality, cardiac implantable electronic device, CIED removal, reinfection, *Staphylococcus aureus* bacteremia

## Abstract

**Background:**

In case of *Staphylococcus aureus* bacteremia (SAB), complete cardiac implantable electronic device (CIED) removal is advised by the European Heart Rhythm Association.

**Objectives:**

The objective of the study was to estimate clinical outcomes after SAB in the Danish CIED carriers.

**Methods:**

We conducted a nationwide register-based cohort study including all patients with SAB after CIED implantation between 2000 and 2020. Cumulative incidence of device removal, SAB reinfection, and all-cause mortality were estimated and compared to sex and age-matched non-CIED controls with SAB. Landmark analysis at the time of hospital discharge estimating mortality and reinfection according to CIED removal status in surviving patients was conducted.

**Results:**

In total, 1,816 patients with CIED and SAB and 9,080 matched controls were included in the study (median age 77.5 years, 73.0% males). Thirty-day all-cause mortality was 34.0% (95% CI: 31.8%-36.2%) in patients with CIED and 31.0% (95% CI: 30.0%-31.9%) in controls (*P* = 0.019, adjusted HR: 1.11 [95% CI: 1.02-1.22]). Device removal within 30 days was performed in 286 patients (15.8%). The landmark analysis showed significantly lower 180-days cumulative incidence of SAB reinfection and all-cause mortality in patients undergoing device removal compared to those with retained CIEDs (reinfection: 2.5% vs 5.5%; mortality: 7.8% vs 31.2%). Patients who underwent CIED removal were younger and had less comorbidity compared to those with retained CIEDs.

**Conclusions:**

The Danish CIED carriers had slightly higher 30-day all-cause mortality after SAB compared to matched controls. Only a minor selected proportion underwent device removal after SAB diagnosis and, after initial survival, these patients had lower 180-days reinfection rates and mortality compared to patients with retained devices.

*Staphylococcus aureus* bacteremia (SAB) is an acute and perilous condition associated with significant morbidity and mortality. Consequently, the mortality rate after SAB has been documented to reach as high as 80% if patients do not receive treatment.^1^ Receiving appropriate antimicrobial treatment significantly decreases mortality; however, approximately 1 in 4 patients will still die as a result of the infection.^2^, ^3^, ^4^, ^5^ The presence of a cardiac implantable electronic device (CIED) could worsen the clinical course of disease due to the risk of device infection. In cases of confirmed CIED infection, complete device removal, in addition to antimicrobial treatment, is recommended.^6^, ^7^, ^8^ However, in cases of systemic infection without definite proof of device involvement the recommendations for device removal differs depending on the encountered microbiological pathogen.^8^ The European Heart Rhythm Association (EHRA) 2020 consensus document advise complete CIED removal in patients with CIED and SAB, regardless of proof of device infection.^8^ However, the European Society of Cardiology (ESC) *2023 guidelines for the management of endocarditis* are adapting a more cautious approach.^6^ However, the current recommendations are largely based on expert opinions, as high-level evidence in this area remains scarce. Device removal is associated with potential serious complications, for example, pericardial effusion, hematoma, hemothorax, valvular, vascular, or structural cardiac damage, and ultimately death.^9^^,^^10^ Consequently, some patients are considered too frail to undergo CIED removal. Therefore, it is crucial to obtain knowledge on current clinical practice and the associated prognosis of patients with CIED and SAB who undergo or do not undergo CIED removal.

In a real-life nationwide cohort of Danish CIED patients with SAB, we aimed to estimate the 30- and 180-day mortality risk and compare the findings to a sex- and age-matched control population with SAB. In addition, we sought to investigate if certain subgroups had an associated higher risk of mortality than others. Lastly, we aimed to estimate the proportion of patients who underwent CIED system removal subsequent to a SAB episode, and to evaluate the association between device removal and clinical outcomes, specifically mortality and risk of reinfection.

## Methods

Using the nationwide Danish registers, we conducted a register-based study cross-linking anonymized health registers and clinical quality databases within the secure facilities of Statistics Denmark. All data were prospectively collected over time.

### Data

A unique and personal identification number is provided to all Danish residents by means of a social security number (Central Person’s Register number) which is used across the entire Danish health care sector and public administration offices to identify residents and to receive public reimbursement for performed services. When anonymized, this number can be used to cross-link the nationwide registers and clinical quality databases on an individual level without compromising patient anonymity and integrity. Through the secure facilities of Statistics Denmark, we used data obtained from the following 5 registers: 1) The National Danish *Staphylococcus aureus* Bacteremia Database hosted by Statens Serum Institut,^11^^,^^12^ holding more than 90% of microbiologically verified SABs in Denmark from 1957 onward; 2) *The Danish Pacemaker and ICD Register,*^13^ which holds information on all CIED-related procedures in Denmark from 1982. The database is continuously updated by the treating physician performing the CIED-procedure; 3) *The Danish Civil Registration System*^14^ which holds information on vital status, sex, migration, date of birth and date of death; 4) *The Danish Register of Medicinal Product Statistics,*^15^ contains information on all prescriptions filled from pharmacies in Denmark; and 5) *The Danish National Patient Register,*^16^ contains data on all hospital admissions and contacts, both in-patient and out-patient, classified according to the International Classification of Diseases (ICD-8/ 10) system.

### Study populations

#### Primary CIED cohort

We identified all adult Danish residents, aged 18 to 99 years with a CIED implantation between January 1, 2000, and December 31, 2020, and subsequent SAB. Index was defined as the date of first positive *S. aureus* blood culture and follow-up continued until the occurrence of endpoint of interest, emigration, or end of study (December 31, 2020), whichever came first. We excluded foreign citizens, patients carrying a leadless device, patients who underwent device removal before the SAB episode, patients who emigrated before CIED implantation, and patients with missing data in the registers, for example, missing data on vital status.

#### Matched control population

As controls, we used a matched population of adult (≥18 years) Danish residents with a positive *S. aureus* blood culture in the study period (January 1, 2000, and December 31, 2020) and no CIED at the time of SAB. CIED patients and controls were matched on sex and age at the time of SAB in a 1 to 5 ratio using propensity score matching based on the nearest-neighbor method. Patients who emigrated before SAB diagnosis and patients with missing data were excluded before matching. Index was defined as the date of first positive *S. aureus* blood culture and follow-up continued until the endpoint of interest, first CIED implantation, death, emigration, or end of study (December 31st, 2020), whichever came first. Patients included in the primary CIED cohort were allowed entry in the control population up until the time of first CIED implantation.

### Clinical characteristics at baseline

Selected clinical comorbidities present at index date were defined according to the primary or secondary diagnosis codes (ICD-8 or ICD-10) related to hospital contacts. Comorbidities were assessed in a clinically relevant timespan before the index date (Supplemental Table 1). In addition, the definitions of diabetes and chronic obstructive pulmonary disease included filled prescriptions for disease-relevant drugs (Supplemental Table 1). Pharmacotherapy at index was defined from minimum 1 redeemed prescription for a given remedy within 6 months before the index date.

### Outcomes

Endpoints of interest included all-cause mortality, CIED removal after SAB, and SAB reinfection. We identified the date of all-cause mortality from *The Danish Civil Registration System,* whereas the date of CIED removal was obtained from *The Danish Pacemaker and ICD Register*. SAB reinfection was identified from *The National Danish Staphylococcus aureus Bacteremia Database* and defined as a *S. aureus* positive blood culture obtained at least 14 days from index SAB.

#### Sensitivity analyses

To apply perspective and evaluate the strength of our results, we conducted a sensitivity analysis estimating the absolute risk of all-cause mortality across age and sex strata in both the primary CIED cohort and the matched control population. In addition, we estimated the hazard of 30-day all-cause mortality in patients with CIED compared to controls using a nonmatched control population consisting of all adult Danish patients with SAB in the study period and no CIED at the time of SAB.

#### Landmark analysis on prognosis according to CIED removal status

We conducted a landmark analysis evaluating the prognosis for all-cause mortality and SAB reinfection according to CIED removal status. To account for potential immortal time bias, we only included patients with CIED and SAB who had a hospital admission related to the SAB episode and who survived to be discharged. Index was defined as the date of discharge following SAB and follow-up continued until SAB reinfection, death, emigration, or end of study (December 31, 2020), whichever came first. The analyses were stratified according to the CIED removal status at the time of hospital discharge.

### Statistical analyses

Clinical characteristics at baseline are presented as crude numbers and percentages for categorical variables and median with IQR for continuous variables. All-cause mortality from the date of SAB was calculated and reported as absolute risk. Reinfection and CIED removal following SAB was calculated and reported as cumulative incidence estimated by the Aalen-Johansen estimator accounting for the competing risk of death. Patients who experienced SAB reinfection or underwent CIED removal were still considered at risk of dying from all causes. Adjusted multivariable Cox proportional hazard regression was used to estimate the HR for 30-day all-cause mortality for patients with CIED compared to matched controls. The following factors were adjusted for: age at the time of SAB, sex, calendar year of SAB diagnosis, ischemic heart disease including revascularization (coronary artery bypass graft/percutaneous coronary intervention), diabetes, cancer, renal dialysis within 6 months of SAB, left-sided valvular disease, alcohol-related hospital contact, liver disease, stroke, chronic obstructive pulmonary disease, systemic corticosteroids, and prior invasive procedures (prosthetic heart valves, surgery [thoracic—not including CIED procedures, abdominal, orthopedic, gynecologic, dermatological, or urologic] within 1 year of SAB). The proportional hazards assumption was tested using scaled Schoenfeld residuals and found valid. Multicollinearity was assessed using corrected generalized variance inflation factors which were all below 2, indicating no multicollinearity among covariates. Interactions between CIED carriage and sex and age, respectively, were tested using the likelihood ratio test.

To identify factors significantly associated with 30-day all-cause mortality within the primary CIED cohort, we constructed an adjusted multivariable Cox model. In addition to the before mentioned covariates, we further evaluated the type of device (pacemaker, implantable cardioverter defibrillator, or cardiac resynchronization therapy device with or without defibrillator capacity), SAB before CIED, and time from CIED implantation to SAB ± 180 days). Factors significantly associated with an increased hazard of 30-day all-cause mortality was used to stratify the primary CIED cohort into groups of low, intermediate, and high risk of mortality, respectively, thus constructing a simplified risk group stratification tool. Patients were awarded 1 point for each of the associated variables present at baseline, and the sum of points was used for risk group stratification. The cutoffs between groups were set at 1 and 2 points, respectively. Finally, we estimated the absolute risk of all-cause mortality according to risk group up until 180 days.

Data analyses and management were carried out through the secure facilities of Statistics Denmark using SAS statistical programme (version 9.4) and R Core Team (2024)—R: A language and environment for statistical computing (R Foundation for Statistical Computing). Data with 3 or fewer observations are reported as ≤3 in accordance with Statistics Denmark’s policy.

### Ethics

In accordance with the Danish Data Protection Act and the General Data Protection Regulation, ethical committee approval or patient consent is not required for Danish register-based studies performed with the sole purpose of statistics and scientific research. Access and approval to use data was granted by the data responsible institute in the Capital Region of Denmark.

## Results

### Study populations and clinical characteristics at baseline

**Figure 1:**
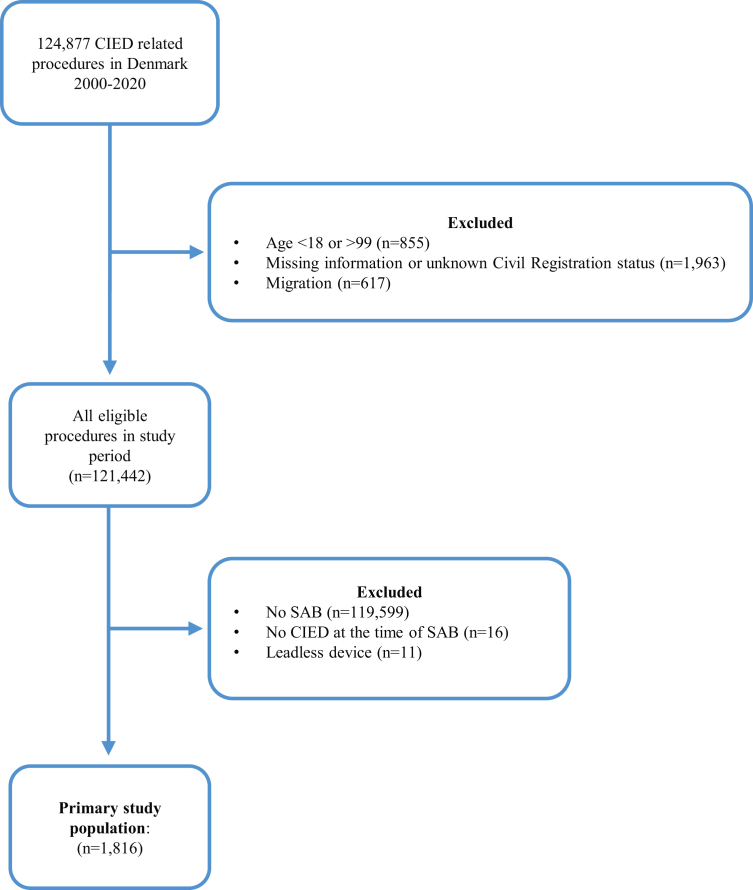
**Flowchart of Study Population** CIED = cardiac implantable electronic device; SAB = *Staphylococcus aureus* bacteremia.

We identified 1,816 patients with a CIED procedure and subsequent SAB in the study period between 2000 and 2020, thus forming the primary CIED cohort (Figure 1). Clinical characteristics at the time of SAB diagnosis are presented in Table 1. The study population comprised 73.0% males with a median age of 77.5 years (IQR: 69-85) at the time of SAB diagnosis. The majority had a pacemaker implanted, whereas the group of patients carrying an implantable cardioverter defibrillator or a cardiac resynchronization therapy device with or without defibrillator capacity was of equal size. The median time from the most recent CIED procedure to SAB diagnosis was 718 days (IQR: 187-1,525 days) and 1,382 patients (76%) experienced the SAB episode following de novo CIED implantation. A total of 1,760 patients had a hospital admission in relation to the SAB episode with a median admission duration of 15 days (IQR 7-30) counting from the date of positive blood culture. A significant proportion of the study population had notable comorbidities (Table 1).

**Table 1:** Clinical Characteristics at Index of the Primary CIED Cohort and Matched Non-CIED Control Population

Clinical Characteristics	Primary CIED Cohort (n = 1,816)	Matched Control Population (n = 9,080)
Demographics		
Age at SAB, y, median [IQR]	77.5 [69, 85]	77.5 [69, 85]
Male, n (%)	1,326 (73.0)	6,634 (73.1)
SAB before CIED, n (%)	64 (3.5)	-
Type of CIED, n (%)		
PM	1,230 (67.7)	-
ICD	302 (16.6)	-
CRT	284 (15.6)	-
Comorbidities, n (%)		
AMI	489 (26.9)	1,126 (12.4)
IHD	1,155 (63.6)	2,679 (29.5)
Congestive heart failure	1,074 (59.1)	1,795 (19.8)
Left-sided valve disease	466 (25.7)	957 (10.5)
Atrial fibrillation	885 (48.7)	2,024 (22.3)
Stroke	461 (25.4)	1,610 (17.7)
COPD	470 (25.9)	1,859 (20.5)
Diabetes	556 (30.6)	1,939 (21.4)
Liver disease	75 (4.1)	585 (6.4)
Cancer	333 (18.3)	2,348 (25.9)
Renal impairment	565 (31.1)	1,825 (20.1)
Hemodialysis within 6 months	165 (9.1)	616 (6.8)
Renal failure	186 (10.2)	653 (7.2)
Alcohol-related hospital contact	74 (4.1)	696 (7.7)
Procedures, n (%)		
Cardiac revascularization	580 (31.9)	965 (10.6)
Prosthetic heart valve	210 (11.6)	293 (3.2)
Surgery within 1 year of SAB	786 (43.3)	3,912 (43.1)
Pharmacotherapy, n (%)		
Anticoagulant therapy	800 (44.1)	1,509 (16.6)
RAAS inhibition	989 (54.5)	3,108 (34.2)
Beta-blockers	1,096 (60.4)	2,753 (30.3)
Lipid lowering agents	866 (47.7)	2,498 (27.5)
Loop diuretics	1,123 (61.8)	3,322 (36.6)
Systemic corticosteroids	243 (13.4)	1,413 (15.6)

AMI = acute myocardial infarction; CIED = Cardiac Implantable Electronic Device; COPD = chronic obstructive pulmonary disease; CRT = cardiac resynchronization therapy w/wo defibrillator; ICD = implantable cardioverter defibrillator; IHD = ischemic heart disease; PM = pacemaker; RAAS = renin-angiotensin-aldosterone system; Cardiac revascularization = Percutaneous coronary intervention or Coronary artery bypass graft surgery; SAB = *Staphylococcus aureus* bacteremia.

#### Matched control population

We identified 9,080 age and sex-matched non-CIED controls with SAB. Clinical characteristics at the time of SAB are presented in Table 1. In general, the matched controls had less cardiovascular comorbidity and received less pharmacotherapy compared to the primary CIED cohort. They did, however, have a higher prevalence of malignant disease compared the CIED cohort. The proportion of patients undergoing surgical interventions (not including CIED-related procedures) within 1 year before SAB diagnosis was equal between the 2 groups.

### Mortality

**Figure 2:**
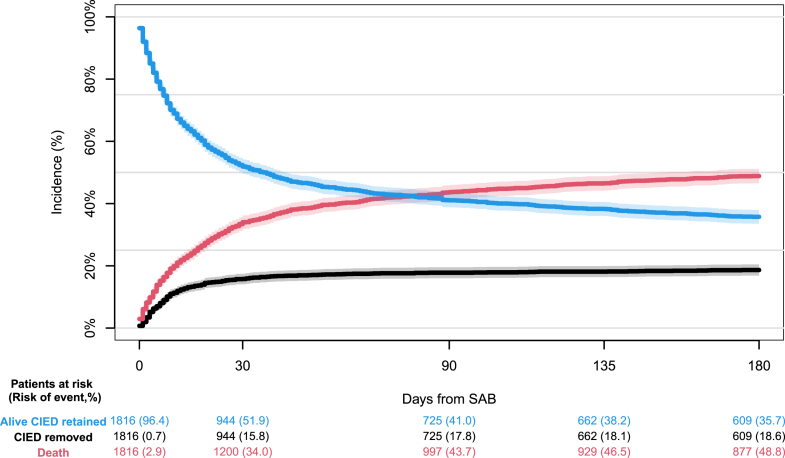
**All-Cause Mortality and CIED Removal** 180 days absolute risk of all-cause mortality, cumulative incidence of CIED removal with competing risk of death, and probability of surviving without device removal in patients with CIED and SAB. Patients who underwent CIED removal were still considered at risk of dying. Abbreviations as in Figure 1.

**Figure 3:**
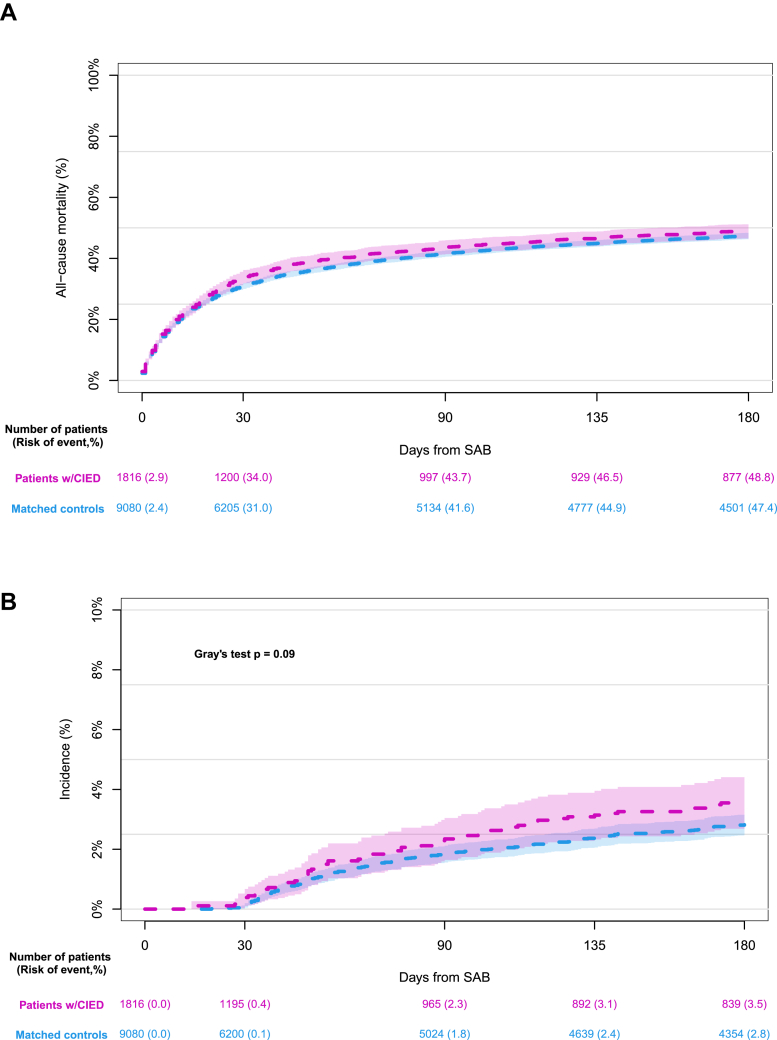
**All-Cause Mortality and Risk of Reinfection** (A) Crude 180 days absolute risk of all-cause mortality following SAB in patients with CIED and controls matched on sex and age at the time of SAB. (B) 180 days cumulative incidence of SAB reinfections accounting for competing risk of death following SAB in patients with CIED and matched controls. Abbreviations as in Figure 1.

Within 30 days of SAB diagnosis, we observed 616 deaths in the primary CIED cohort corresponding to a 30-day all-cause mortality of 34.0% (95% CI: 31.8-36.2) (Figures 2 and 3A, Central Illustration). Among controls 2,801 patients died during the same time span equivalent to a 30-day all-cause mortality of 31.0% (95% CI: 30.0-31.9) (*P* = 0.019) (Figure 3A). The fully adjusted analysis demonstrated a slightly, increased hazard of 30-day all-cause mortality among patients with CIED compared to matched controls (HR: 1.11; 95% CI: 1.02-1.22; *P* = 0.022). No interactions between CIED and age (*P* = 0.457) or sex (*P* = 0.601) were observed.

**Central Illustration:**
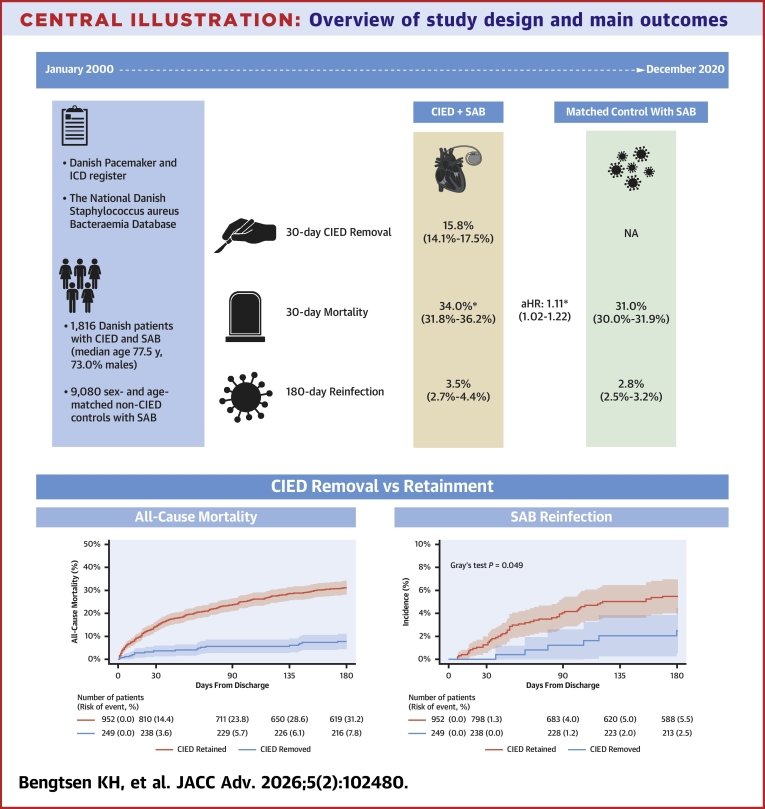
**Overview of study design and main outcomes** Identified from Danish nationwide registers, 1,816 patients with CIED and SAB were compared to 9,080 age- and sex-matched controls with SAB but no CIED. Lower panel: Landmark analysis on prognosis stratified according to CIED removal status at the time of hospital discharge. (Left) Absolute risk of all-cause mortality at 180 days following SAB. (Right) Cumulative incidence of SAB reinfections 180 days accounting for competing risk of death. ∗*P* < 0.05. Created in BioRender. Bengtsen K (2025), https://BioRender.com/sn9fitx. CIED = cardiac implantable electronic device; SAB = *Staphylococcus aureus* bacteremia; ICD = implantable cardioverter defibrillator.

At 180 days from SAB diagnosis, we observed no difference in mortality between patients with CIED and controls, 48.8% vs 47.4% (*P* = 0.171).

The sensitivity analysis comparing mortality between CIED patients and matched controls in age and sex strata showed no difference at neither 30 nor 180 days (Supplemental Table 2). Using an unmatched control population consisting of all Danish patients with SAB without CIED in the study period (n = 27,474; median age 69 years [IQR: 58-79], clinical characteristics at the time of SAB available online) (Supplemental Table 3) revealed results similar to the main analysis producing an adjusted 30-day all-cause mortality HR of 1.16 (95% CI: 1.07-1.27) for patients with CIED compared to controls (30-day absolute risk of all-cause mortality in unmatched controls 26.2% [95% CI: 24.4-25.5]).

#### Factors associated with increased hazard of mortality and risk group stratification

**Figure 4:**
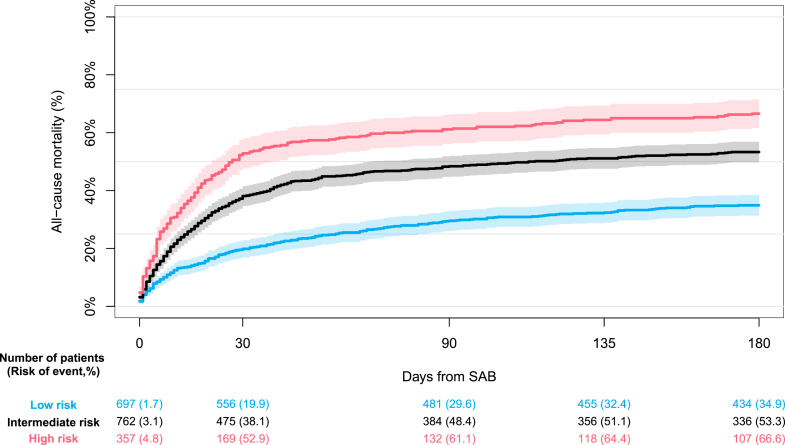
**Risk Group Stratification** Absolute risk of 180-day all-cause mortality according to risk groups. Patients were awarded 1 point for each of the following conditions present: Age above 75 years, SAB occurring >6 months from most recent CIED implantation, left-sided valvular disease, alcohol-related hospital contact, and the use of systemic corticosteroids. The sum of points was used for risk group stratification. Cutoffs between groups were set at 1 and 2 points. Abbreviations as in Figure 1.

From the multivariable model in the primary CIED cohort we observed age above 75 years, SAB occurring more than 6 months from CIED implantation, left-sided valvular disease, alcohol-related hospital contact, and the use of systemic corticosteroids to be significantly associated with an increased hazard of 30-day all-cause mortality (Supplemental Figure 1). Univariate analyses of the covariates and the risk group stratification tool are available online (Supplemental Tables 4 and 5). The median total score obtained after risk group stratification was 2 points (IQR: 1-2) corresponding to 697, 762, and 357 patients in the low, intermediate, and high-risk groups, respectively. The absolute risk of all-cause mortality according to risk groups is presented in Figure 4. The high-risk group showed a significantly higher risk of dying from all causes compared to the low-risk group at both 30 days (52.9% vs 19.9%) and 180 days from SAB (66.6% vs 34.9%).

### SAB reinfection

Within 180 days of index SAB, 63 of 1,816 patients with CIED and 248 of 9,080 matched controls experienced a SAB reinfection corresponding to 180-days cumulative incidences of 3.5% (95% CI: 2.7%-4.4%) and 2.8% (95% CI: 2.5%-3.2%), (*P* = 0.095), respectively, accounting for competing risk of death (Figure 3B).

### CIED removal

In the primary CIED cohort, 286 patients underwent CIED removal within 30 days of SAB diagnosis equivalent to a cumulative incidence of 15.8% (95% CI: 14.1%-17.5%) accounting for competing risk of death (Figure 2). Of the patients who underwent device removal, 69.9% (n = 200) had the procedure undertaken within the first 10 days of SAB diagnosis. Less than or equal to 3 patients died the same day as the CIED was removed. Nine hundred forty-four patients (51.9%) were alive 30 days from SAB diagnosis without having undergone CIED removal.

### Landmark analysis on prognosis according to CIED removal status

Of the 1,760 patients in the primary CIED cohort who had a hospital admission related to their SAB episode, 1,201 were alive to be discharged (Supplemental Figure 2). Two hundred forty-nine (20.7%) of these patients underwent CIED removal during the SAB admission. Of these, 172 (69.1%) patients had a diagnosis of infective endocarditis (IE) during SAB hospitalization compared to 128 patients (13.4%) in the group with retained CIED. The median time to CIED removal from the date of SAB diagnosis was 6 days (IQR: 3-11). The group of patients who underwent CIED removal were significantly younger than the group who had their device retained (70 vs 77 years old) and had less ischemic heart disease, less impaired renal function, and less dialysis treatment. Apart from these factors, the groups were comparable (Table 2).

**Table 2:** Clinical Characteristics at the Time of Hospital Discharge of the Primary CIED Cohort According to CIED Removal Status

Clinical Characteristics	CIED Retained (n = 952)	CIED Removed (n = 249)	Total (N = 1,201)
Demographics			
Age at SAB, y, median [IQR]	77 [70, 84]	70 [59, 78]	76 [67, 83]
Male, n (%)	704 (73.9)	199 (79.9)	903 (75.2)
Previous SAB n (%)	36 (3.8)	8 (3.2)	44 (3.7)
Type of CIED, n (%)			
PM	646 (67.9)	138 (55.4)	784 (65.3)
ICD	148 (15.5)	74 (29.7)	222 (18.5)
CRT	158 (16.6)	37 (14.9)	195 (16.2)
Comorbidities, n (%)			
AMI	269 (28.3)	76 (30.5)	345 (28.7)
IHD	629 (66.1)	145 (58.2)	774 (64.4)
Congestive heart failure	561 (58.9)	138 (55.4)	699 (58.2)
Left-sided valve disease	217 (22.8)	55 (22.1)	272 (22.6)
Atrial fibrillation	490 (51.5)	99 (39.8)	589 (49.0)
Stroke	238 (25.0)	63 (25.3)	301 (25.1)
COPD	245 (25.7)	52 (20.9)	297 (24.7)
Diabetes	311 (32.7)	79 (31.7)	390 (32.5)
Liver disease	45 (4.7)	6 (2.4)	51 (4.2)
Cancer	176 (18.5)	35 (14.1)	211 (17.6)
Renal impairment	335 (35.2)	47 (18.9)	382 (31.8)
Hemodialysis within 6 months	111 (11.7)	17 (6.8)	128 (10.7)
Renal failure	126 (13.2)	18 (7.2)	144 (12.0)
Alcohol-related hospital contact	40 (4.2)	8 (3.2)	48 (4.0)
IE during SAB hospitalization	128 (13.4)	172 (69.1)	300 (25.0)
Procedures, n (%)			
Cardiac revascularization	314 (33.0)	94 (37.8)	408 (34.0)
Prosthetic heart valve	93 (9.8)	28 (11.2)	121 (10.1)
Any surgery within 1 y	499 (52.4)	139 (55.8)	638 (53.1)
Pharmacotherapy, n (%)			
Anticoagulant therapy n (%)	416 (43.7)	98 (39.4)	514 (42.8)
RAAS inhibition n (%)	518 (54.4)	147 (59.0)	665 (55.4)
Beta-blockers n (%)	566 (59.5)	151 (60.6)	717 (59.7)
Lipid lowering agents n (%)	445 (46.7)	140 (56.2)	585 (48.7)
Loop diuretics n (%)	592 (62.2)	116 (46.6)	708 (59.0)
Systemic corticosteroids n (%)	109 (11.4)	20 (8.0)	129 (10.7)

Abbreviations as in Table 1.

We observed a more than 3-fold higher absolute mortality risk within 180 days of hospital discharge when comparing the group of patients with retained CIEDs to those who had CIED removal performed during the admission related to SAB (31.2% vs 7.8%; adjusted HR for CIED removal: 0.30; 95% CI: 0.18-0.48; *P* < 0.001) (Central Illustration). The highest mortality rate was observed within the first 30 days of discharge in the group with retained CIED. Similarly, we observed a significantly higher risk of SAB reinfection at 180 days from index SAB among patients with retained CIED compared to those who underwent CIED removal: 5.5% (95% CI: 4.0%-6.9%) vs 2.5% (95% CI: 0.5%-4.4%), *P* = 0.049 (Central Illustration). Adjusted analysis revealed a 180-day cause specific HR of 0.49 (95% CI: 0.20-1.20) for SAB reinfection with CIED removal compared to CIED retainment.

Five years from discharge, the absolute risk of dying was still significantly increased in the group of patients who did not undergo device removal compared to those who did (Supplemental Figure 3A). However, the risk of SAB reinfection equalized between the 2 groups over time (Supplemental Figure 3B).

## Discussion

The main finding from our study was a crude 30-day all-cause mortality of 34.0% increasing to 48.8% at 180 days from SAB diagnosis among patients with CIED. Compared to matched non-CIED controls with SAB, the mortality among patients with CIED was slightly higher at 30 days, but not at 180 days after SAB. Furthermore, among the CIED patients who survived until hospital discharge, we observed lower mortality and SAB reinfection rates at 180 days from discharge among patients who underwent CIED removal compared to those with retained CIEDs.

Bai et al.^4^ conducted a systematic review of 341 worldwide studies investigating the mortality following SAB in more than 500,000 unselected patients. Between January 1, 1991, and May 7, 2021, they observed a total all-cause mortality of 19.6% (18.4% to 20.9%) and 27.4% (24.1% to 30.9%) at 1 and 3 months, respectively. In a Dutch multicenter prospective cohort (n = 490), van der Vaart et al.^5^ observed a 30- and 90-day all-cause mortality after SAB of 23.7% and 32.9%, respectively. Berge et al.^17^ analyzed a Swedish cohort of patients with CIED and SAB and observed an overall 30-day mortality of 28% (n = 274, median age 82 years) and thus roughly comparable to ours. Danish data by Mejer et al.^3^ reported an overall 30-day mortality from SAB of 25.7% observed from 16,330 SAB cases in the general population through the years 1995 to 2008. These reports of approximately 1 in 4 patients with SAB will die within 30 days underscores the serious and dangerous nature of the condition. In our selected patient cohort carrying a CIED, we observed a 30% to 70% relatively higher risk of all-cause mortality at 30 days compared to the previously mentioned unselected cohorts and thus one might wonder if the CIED itself poses a risk of a worse outcome following SAB. When comparing the crude mortality rates, it is important to pay attention to the fact that our CIED cohort had a high median age, and a substantial proportion of the cohort had notable comorbidities, including congestive heart failure, prior myocardial infarction, and the underlying conditions giving rise to the CIED indication. The patients in the cohort examined by van der Vaart et al.^5^ had a median age of 68 years (IQR: 57-77) and thus, the median age was almost 10 years lower compared to the patients in our cohort. Mejer et al.^3^ did not report a median age of the overall cohort, but approximately 1/3 of both community-acquired and hospital-associated SAB episodes were observed in patients over the age of 75 years. They did, however, report an age stratified 30-day mortality proportion ranging between 0.37 (2007-2008) and 0.45 (1995-1997) in patients older than 75 years and between 0.23 and 0.29 in patients aged 36 to 55 years. Compared to the findings of Mejer et al., our results suggest that patients with a CIED carry a slightly higher mortality risk following a SAB episode compared to the general population.

Our observations of higher 30-day mortality among patients with CIEDs compared to matched controls may be partly explained by a greater comorbidity burden in the CIED group. However, the difference persisted after statistical adjustment for potential confounders, suggesting that the presence of an implanted foreign body with vascular access may complicate the clinical course of SAB. Assessed at 180 days from index SAB, we observed no mortality difference between patients with CIED and controls thus indicating the foreign body contribution may be most critical in terms of short-term mortality. Even though we adjusted for potential confounders we cannot exclude the possibility of residual confounding and thus the results must be interpreted with caution.

When considering reinfection patterns in our cohorts, the rates were similar to previous studies.^18^, ^19^, ^20^, ^21^ Given equal reinfection rates between patients with CIED and matched controls and that most reinfections occurred more than 30 days from index SAB, it does not explain the higher 30-day mortality observed among the patients with CIED.

### Risk group stratification

After patient stratification into risk groups, we observed a significantly increased risk of all-cause mortality in the intermediate- and high-risk groups compared to the low-risk group. The analysis highlights the risk differences according to predisposing comorbidity at baseline and underscores the need for careful individual evaluation of every patient. The stratification tool is constructed with the purpose of illustrating the risk difference across the cohort and is not intended for clinical use for which it is deficient. To apply a stratification tool in clinical practice, information about the infection and course of disease (eg device involvement, embolization, valve endocarditis, intensive care treatment etc.) would need incorporation in the model, information which unfortunately is not available in the registers. Furthermore, the generalizability of the model should be validated in another cohort.

### Device removal

In our primary CIED cohort, 286 (15.8%) patients underwent device removal within 30 days of SAB diagnosis, with the majority being extracted within 10 days (69%). Removal rates following SAB have previously been estimated to 12% to 36% across all patients irrespective of confirmed device infection.^17^^,^^22^^,^^23^ From the data contained in the used registers, we are not able to determine if the patients in our cohort had device involvement concomitant to the bacteremia, which would require imaging and/or autopsy data. However, from the landmark analysis, we observed that 69.1% of the patients who underwent CIED removal had an IE diagnosis during index hospitalization, thus representing an intracardiac infection. Although CIED removal is a class I recommendation in patients with definite device infection, the *2023 ESC Guidelines for the Management of Endocarditis* also recommend CIED removal in cases of prolonged or relapsing bacteremia, even when there are no signs of device involvement.^6^ The 2023 American Heart Association (AHA)’s *Update on Cardiovascular Implantable Electronic Device Infections* recommends complete device removal in the case of 2 positive blood cultures and echocardiographic signs of device involvement or in the case of prolonged bacteremia.^7^ In contrast, the 2020 EHRA consensus document on CIED infections advice complete CIED removal in the case of SAB regardless of echocardiographic findings.^8^ Unfortunately, we were not able to determine how many patients were examined by a cardiologist or had an echocardiography performed, an examination which is the recommended first-line imaging modality when suspecting endocarditis and device infections. However, the observed removal pattern does not comply with the contemporary recommendations from EHRA which would warrant removal proportions close to 1. Given the risk of device infection in patients with CIED and SAB is reported as high as 50%,^22^^,^^24^ one would expect that roughly 3 times as many patients from our cohort should have undergone device removal if the ESC and AHA recommendations were to be followed. Importantly, other studies have observed lower risk of device infection in the presence of SAB,^17^^,^^23^^,^^25^ and consequently the mismatch between our observed removal rates and the rates expected from the guideline-recommendations could be smaller. It is important to emphasize that the examined CIED cohort precedes the 2020 EHRA, 2023 ESC, and 2023 AHA guidelines in time. However, the 2015 ESC *Guidelines for the management of infective endocarditis**^26^* recommended complete device removal in the case of definite device infection and further to consider removal in cases of suspected device infection or occult infection without any apparent source. The observation of low removal rates in our cohort probably reflects different practice patterns across treatment facilities, evolving CIED removal trends over time, individualized treatment regimens with considerations on periprocedural risks, for example, hematoma, pneumothorax or hemothorax, valvular-, vascular-, or structural cardiac damage, and death. In addition, it is possible that a proportion of patients never encountered a cardiologist, and thus never got evaluated for CIED removal.

### Landmark analysis of prognosis according to CIED removal status

In the landmark analysis of all CIED patients who survived until hospital discharge, we observed a significantly higher 180-days cumulative incidence of SAB reinfection in patients with retained CIEDs compared to patients who underwent CIED removal during SAB hospitalization. These findings may imply that CIED retainment in relation to SAB constitutes a risk factor for reinfection by virtue of the presence of implanted foreign material with vascular access. However, over time, the cumulative incidence of SAB reinfections equalized between the groups indicating that any additional risk with CIED retainment compared to CIED removal would be most prevalent within the first year. The adjusted analysis showed no significant association between CIED removal and lower hazards of SAB reinfection but did however lack power to draw a definite conclusion.

We observed a significantly higher mortality (short and long term) among patients with retained CIEDs compared to those who underwent device removal during hospitalization. It is important to note that the groups differed significantly with respect to age and selected comorbidities highlighted by the group undergoing CIED removal, being significantly younger than the group with retained devices. Thus, one would expect a higher age-related mortality in the group with retained CIEDs. Furthermore, 69.1% of the patients in the CIED removal group had a diagnosis of IE during the SAB hospitalization compared to 13.4% among the patients with retained CIED. These observations, in addition to unknown factors like frailty and severity of illness, may have affected both mortality and the decision to remove the CIED. The analyses have an inherent risk of confounding by indication given that older, frail patients with more chronic comorbidity are in increased risk of complications related to the removal procedure and thus might be less likely to undergo device removal.^27^, ^28^, ^29^ With these limitations in mind, the results should be interpreted with caution. Nevertheless, the adjusted analysis showed a significant association between CIED removal and lower hazards of all-cause mortality. Berge et al^30^ assessed the 30-day and 1-year mortality from SAB according to device removal status in a regional Swedish cohort of CIED patients (n = 274) and observed an increased mortality among the patients with retained devices at 30 days, but no difference at 1 year (*P* = 0 0.11). In a sensitivity analysis, they excluded all patients who died within the first 14 days resulting in no difference in mortality at 30-day follow-up. However, the authors did not explain in detail about the time aspect of the CIED removal classification related to the index point and thus the risk of immortal time bias might not be accounted for. Other studies have compared the mortality between patients who underwent device removal and patients with retained devices following definite device infection and found a beneficial effect of device removal. Athan et al.^31^ observed the 1-year mortality among 175 patients with CIED infection to be 19.9% in patients who had their device removed during index hospitalization compared to 38.2% in patients with retained devices. Likewise, Le et al.^32^ observed an adjusted HR of 6.97 (95% CI: 1.36-35.60) of dying when treated with antibiotics alone compared to antibiotics in combination with complete device removal (n = 416). Pokorney et al.^33^ evaluated the 1-year mortality of 10,832 CIED infections according to removal status at 30 days from positive blood culture and found an adjusted HR of 0.82 (95% CI: 0.74-0.90) with removal compared to no removal. The embedded limitations of these observational studies call for randomized clinical trials to further explore the subject.

### Strengths and limitations

Observational studies come with inherent limitations including the lack of possibilities of revealing causal relationships and thus results should be interpreted with caution. We consider the size and nationwide coverage of the present study a great strength. Furthermore, the registers used in the study are considered of high quality and with virtually complete coverage during the study years and thus we consider the risk of potential selection bias minimal. Among the limitations are missing information on clinical parameters and imaging results which again leads to lack of possibilities of determining if the included patients had device involvement or merely bacteremia with bystander CIED. Specifically, we are not able to determine if the patients who had their device removed had definite CIED infection. The proportion of patients with a diagnosis code for IE could be interpreted as an indicator of CIED infection, but as the diagnosis code is neither specific nor validated for this use, it should be approached with caution. As touched on in the discussion we cannot exclude the possibility of confounding by indication given that older and more fragile patients are at an increased risk of complications related to the device removal procedure and thus might be more likely to have the CIED retained. Although we included relevant potential confounders in our adjusted analyses, the risk of residual confounding cannot be excluded.

## Conclusions

In this nationwide observational study, patients with SAB and a concomitant CIED had an increased 30-day mortality compared to matched SAB patients without a CIED. Although CIED extraction was undertaken in only a minority of cases, among patients surviving the index hospitalization, device removal was associated with reduced 180-day all-cause mortality and SAB reinfection. These findings highlight the importance of rigorous diagnostic evaluation, timely treatment decisions, and structured follow-up of patients with CIED who develop SAB.

## Funding support and author disclosures

Arvid Nilsson’s Foundation and Health Insurance Denmark (Sundhedsdonationer: Grant number 2024-0509) supported this work with salary covering grants to the main author Dr Bengtsen. Dr Ruwald reports receiving honoraria for educational presentations not related to the current study from Abbott, Novartis, and Bristol Meyer Squibb and she reports receiving travel support and payment of congress fee not related to the current study from Pharmacosmos. Dr Torp-Pedersen reports receiving grants for studies from Novo Nordisk and Bayer not related to the current study. Dr Fosbøl reports independent and unrelated research grants from the 10.13039/501100009708Novo Nordisk Foundation and 10.13039/100007405Danish Heart Association for valvular heart disease research. Dr Nielsen reports a role as executive editor for Europace. Dr Østergaard reports receiving an independent research grant from the 10.13039/501100009708Novo Nordisk Foundation not related to the current study. Dr Bruun reports receiving investigator-initiated grants from the 10.13039/501100009708Novo Nordisk Foundation, the 10.13039/501100004954Augustinus Foundation, the Kaj Hansen Foundation, and Health Insurance Denmark, not related to the current study. All other authors have reported that they have no relationships relevant to the contents of this paper to disclose.
