# Supplementary material for: Device-Removal, Reinfection, and Mortality After Staphylococcus aureus Bacteremia in Patients With Cardiac Implantable Electronic Devices
Source: JACC Adv. 2026 Jan 20;5(2):102480. doi: 10.1016/j.jacadv.2025.102480 (PMC12856339; doi:10.1016/j.jacadv.2025.102480)
Supplement: Supplemental_Material [file mmc1.pdf]

Supplementary Material

For

Bengtsen et al.

Device-Removal, Reinfection and Mortality after *Staphylococcus aureus* Bacteraemia in Patients with Cardiac Implantable Electronic Devices

Content

- Table S1: ICD, NOMESCO and ATC codes.
- Table S2: Stratified risk of all-cause mortality
- Table S3: Clinical characteristics at index of the sensitivity non-matched control population comprising all patients with SAB in the study period but without CIED.
- Table S4: Univariate cox analysis of covariates associated with 30-day all-cause mortality from date of SAB diagnosis
- Table S5: Risk-group stratification tool comprising clinical variables and corresponding adjusted hazard ratios of factors associated with 30-day all-cause mortality after SAB in patients with CIED.
- Figure S1: Forest plot of adjusted Cox model of covariates associated with 30-day all-cause mortality
- Figure S2: Flowchart of the population included in the landmark analysis conducted at the time of hospital discharge.
- Figure S3 A+B: Landmark analysis at the time of hospital discharge of 5-year absolute risk of all-cause mortality (A) and 180-days cumulative incidence of reinfections (B).

Table S1. ICD, NOMESCO and ATC codes.

	ICD-10	ICD-8	NOMESCO	ATC	Time evaluated prior to SAB
**Comorbidity**					
**AMI**	DI21-22				All time
**IHD**	DI20, 23,24,25	410-414	KFNG, KFNA, KFNB, KNFC, LFND, KFNE		All time
**Congestive heart failure**	DI50, DI110, DI130, DI132, DI420, DI426, DI427, DI428, DI429	425, 428			All time
**Left-sided valve disease**	DI35, DI34	394, 395, 396			All time
**Atrial fibrillation**	DI48				5 Y
**Stroke**	DI 60, DI61, DI63, DI64, DG45	430-436			All time
**COPD**	DJ43-44	490-492		R03BA, R03AK06-R03AK12, R03AL08, R03AL09, R03AC, R03AL, R03BB	5 Y
**Diabetes**	DE10-14, DO24 (DO244 excluded), DH360	249, 250		A10	5 Y
**Liver disease**	DB15-19, DC22, DK70-77, DI85, DI982, DZ944, DQ618A				5 Y
**Cancer**	DC00-97	140-209			5 Y
**Renal impairment**	DE102, DE112, DE132, DE142, DI120, DM300, DM313, DN158-160, DN162-164, DN168	403, 404, 581-584			5 Y
**Haemodialysis within 6 months**	DZ992		BJFD2		6 M
**Renal failure**	DN185, DZ992				5 Y
**Alcohol-related hospital contact**	DF10, DK70, DT51, DI426, DG621, DG721, DK292, DE244, DK852, DK860, DG312, DZ721, DZ14, DL278A, DE529A				5 Y

**Infective endocarditis during SAB hospitalization**	DI33, DI38, DI398	421			During SAB hospitalization
**Procedures**					
**Revascularization**			KFNG, KFNA, KFNB, KNFC, LFND, KFNE		All time
**Prosthetic heart valve**			KFKD, KFMD, KFGE, KFJF		All time
**Surgery within 1 year**			KF, KG, KJ, KK, KL, KN, KQ		1 Y
**Pharmacotherapy**					
**Anticoagulant therapy**				B01AA03, B01AA04, B01AE07, B01AF	6 M
**RAAS inhibition**				C09	6 M
**Beta-blockers**				C07	6 M
**Lipid lowering**				C10	6 M
**Loop diuretics**				C03C	6 M
**Systemic corticosteroids**				H02	6 M

Table S2. Absolute risk of all-cause mortality at 30 and 180 days from positive *Staphylococcus aureus* blood culture in the primary CIED cohort and age and sex matched controls with SAB but without CIED. Absolute risk of all-cause mortality % (95% Confidence interval).

All-cause mortality from date of SAB 2000-2020

	**30 days**		**180 days**	
	Primary CIED cohort n= 1,816	Matched control population n= 9,080	Primary CIED cohort n= 1,816	Matched control population n= 9,080
**Overall**	34.0 (31.8-36.2)*	31.0 (30.0-31.9)	48.8 (46.5-51.2)	47.4 (46.3-48.4)
**Age (years)**				
18-65	19.0 (14.8-23.3)	15.9 (14.1-17.7)	30.7 (25.7-35.7)	27.8 (25.7-30.0)
66-75	24.4 (20.5-28.3)	23.1 (21.4-24.8)	43.8 (39.3-48.4)	39.6 (37.6-41.7)
76-85	38.3 (34.5-42.2)	34.2 (32.6-35.9)	50.7 (46.8-54.6)	51.7 (50.0-53.5)
>85	51.3 (46.4-56.3)	48.0 (45.8-50.3)	67.5 (62.7-72.2)	66.3 (64.2-68.4)
**Sex**				
Male	31.7 (29.2-34.2)	29.4 (28.3-30.5)	46.8 (44.1-49.5)	45.8 (44.6-47.0)
Female	40.3 (35.9-44.6)	35.4 (33.5-37.3)	54.5 (50.0-58.9)	51.6 (49.6-53.6)

Abbreviations: CIED, Cardiac Implantable Electronic Device; SAB, *Staphylococcus aureus* bacteraemia. * Indicates Significant values ($p < 0.05$) compared to the matched control population.

Table S3 Clinical characteristics at index of the sensitivity non-matched control population comprising all patients with SAB in the study period but without CIED.

	**Sensitivity control population N= 26,727**
**Demographics**	
Age at SAB, y, median [IQR]	69 [57, 79]
Male sex	16,312 (61.0)
**Comorbidities**	
AMI	2.519 (9.4)
IHD	6,269 (23.5)
Congestive heart failure	4,091 (15.3)
Left-sided valve disease	2,178 (8.1)
Atrial fibrillation	4,375 (16.4)
Stroke	3,931 (14.7)
COPD	5,105 (19.1)
Diabetes	5,592 (20.9)
Liver disease	2,526 (9.5)
Cancer	6,892 (25.8)
Renal impairment	5,351 (20.0)
Hemodialysis within 6 months	2,227 (8.3)
Renal failure	2,347 (8.8)
Excessive alcohol consumption	2,959 (11.1)
**Procedures**	
Re-vascularization	2,237 (8.4)
Prosthetic heart valve	668 (2.5)
Surgery within 1 year of SAB	11,830 (44.3)
**Pharmacotherapy**	
Anticoagulant therapy	3,452 (12.9)
RAAS inhibition	8,006 (30.0)
Beta-blockers	7,116 (26.6)
Lipid lowering agents	6,469 (24.2)
Loop diuretics	8,533 (31.9)
Systemic corticosteroids	4,031 (15.1)

Abbreviations: AMI, acute myocardial infarction; CIED, Cardiac Implantable Electronic Device; COPD, chronic obstructive pulmonary disease; IHD, ischemic heart disease; IQR, interquartile range; RAAS, renin-angiotensin-aldosterone system; SAB, *Staphylococcus aureus* bacteraemia.

Table S4 Univariate cox analysis of covariates associated with 30-day all-cause mortality from date of SAB diagnosis

	**HR**	**Lower**	**Higher**	**P-value**
**Sex**				
Female	1			
Male	0.74	0.63	0.88	<0.001
**Age (years)**				
18-65	1			
66-75	1.33	0.97	1.80	0.073
76-85	2.28	1.73	3.01	<0.001
>85	3.38	2.54	4.49	<0.001
**Device type**				
PM	1			
ICD	0.62	0.49	0.80	<0.001
CRT	0.82	0.65	1.03	0.088
**Implantation year**				
2016-2020	1			
2012-2015	1.02	0.84	1.24	0.865
2008-2011	0.84	0.66	1.06	0.148
2004-2007	0.82	0.62	1.08	0.156
2000-2003	1.00	0.69	1.46	0.998
**Comorbidity**				
Ischemic Heart disease	0.95	0.81	1.12	0.534
Surgery within 1 year	1.10	0.94	1.29	0.239
Cancer	1.29	1.07	1.57	0.008
Left-sided valve disease	1.50	1.27	1.78	<0.001
Prosthetic heart valves	1.45	1.16	1.81	0.001
Alcohol-related hospital contact	1.18	0.81	1.72	0.398
Systemic corticosteroids	1.53	1.25	1.88	<0.001
Diabetes	0.86	0.72	1.02	0.089
Liver disease	1.27	0.89	1.82	0.194
Stroke	1.21	1.02	1.44	0.032
COPD	1.11	0.93	1.32	0.252
Dialysis w/in 6 months	0.78	0.58	1.05	0.102
Previous SAB	0.83	0.53	1.32	0.439
SAB > 6 months from CIED implantation	1.64	1.33	2.02	<0.001

Table S5 Risk-group stratification tool comprising clinical variables and corresponding adjusted hazard ratios of factors associated with 30-day all-cause mortality after SAB in patients with CIED.

Clinical variable	β	SE	Hazard ratio (95% CI)	Point awarded if condition present
Age 18 - 65 years			Reference	0
Age 66 - 75 years	0.23	0.16	1.25 (0.92-1.72)	0
Age 76 - 85 years	0.82	0.15	2.26 (1.68-3.05)	1
Age >85 years	1.24	0.16	3.45 (2.50-4.76)	1
Left-sided valvular disease	0.33	0.11	1.39 (1.12-1.72)	1
Alcohol-related hospital contact	0.49	0.22	1.63 (1.05-2.53)	1
SAB > 6 months from CIED implantation	0.40	0.11	1.48 (1.20-1.84)	1
Systemic corticosteroids	0.38	0.11	1.47 (1.18-1.81)	1

Abbreviations: CIED, Cardiac Implantable Electronic Device; SAB, Staphylococcus aureus bacteraemia. SE: Standard Error.

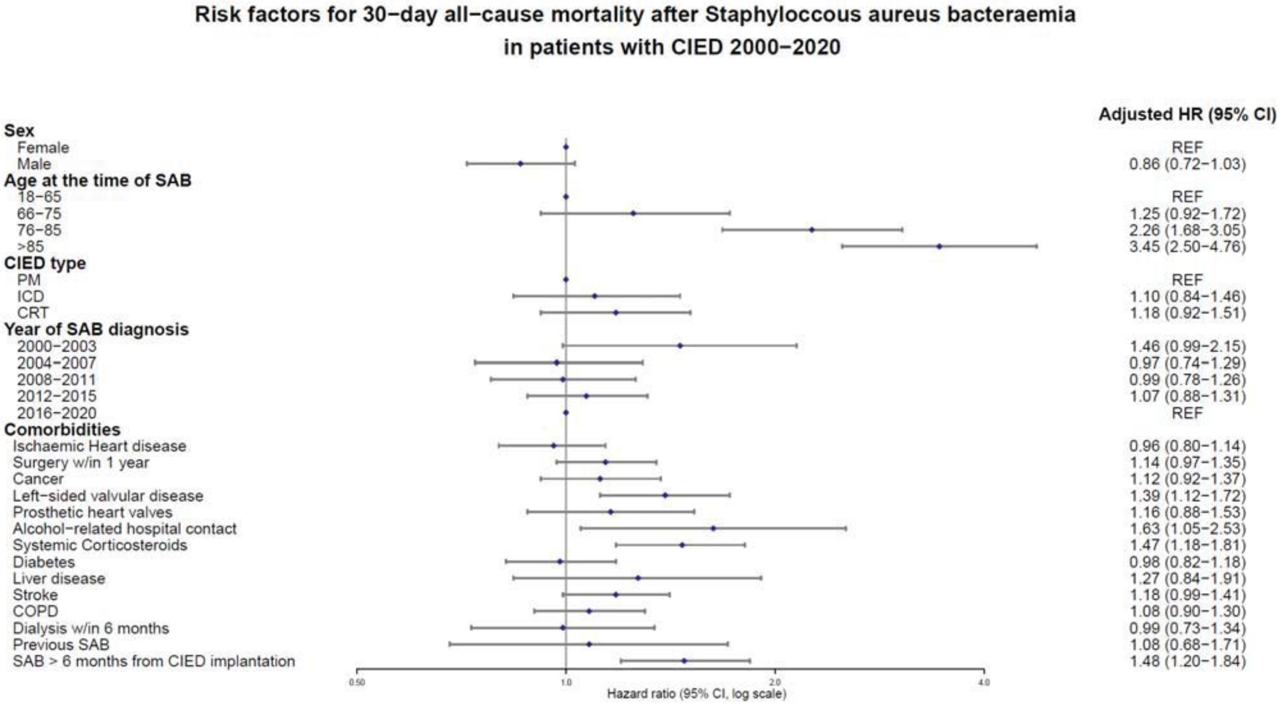

Figure S1

Forest plot of adjusted Cox model of covariates associated with 30-day all-cause mortality from date of Staphylococcus aureus bacteraemia in patients with cardiac implantable electronic device, CI: Confidence interval, COPD: Chronic obstructive pulmonary disease, CRT: Cardiac resynchronization therapy device w/wo defibrillator capacity, HR: Hazard ratio, ICD: Implantable cardioverter defibrillator, PM: Pacemaker; SAB: Staphylococcus aureus bacteraemia

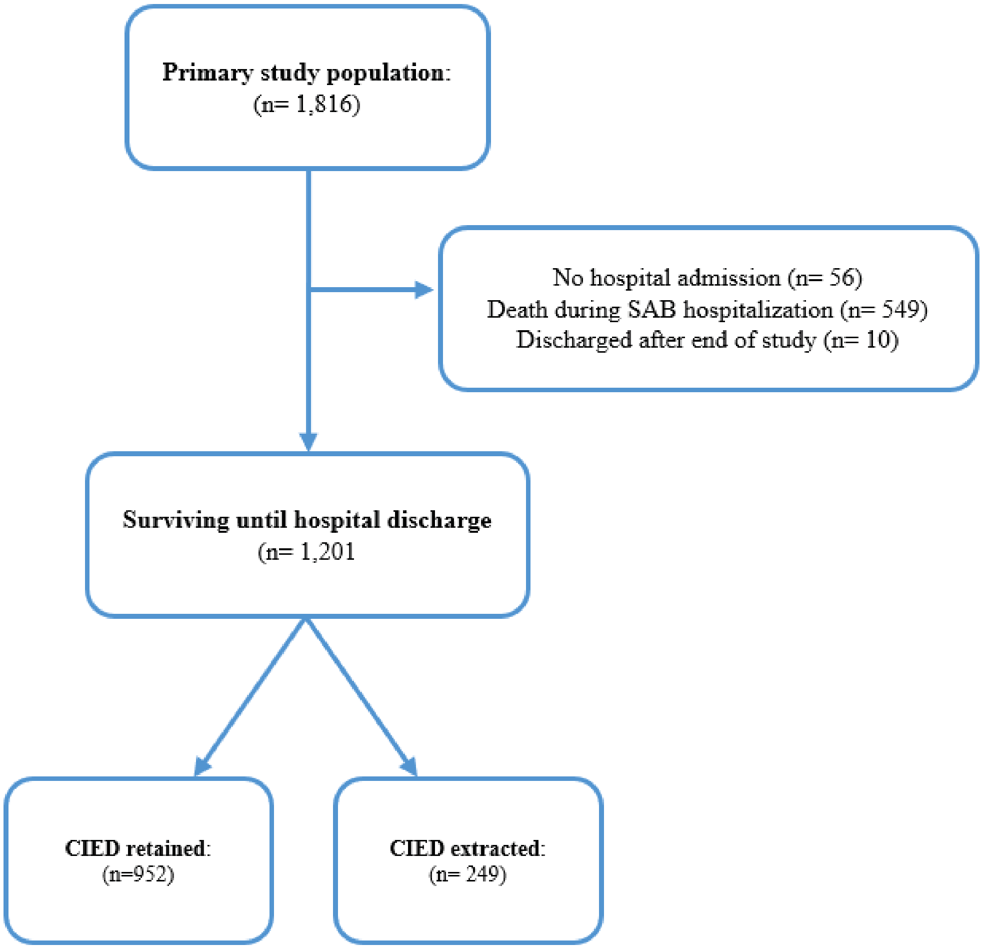

Figure S2: Flowchart of the population included in the landmark analysis conducted at the time of hospital discharge.

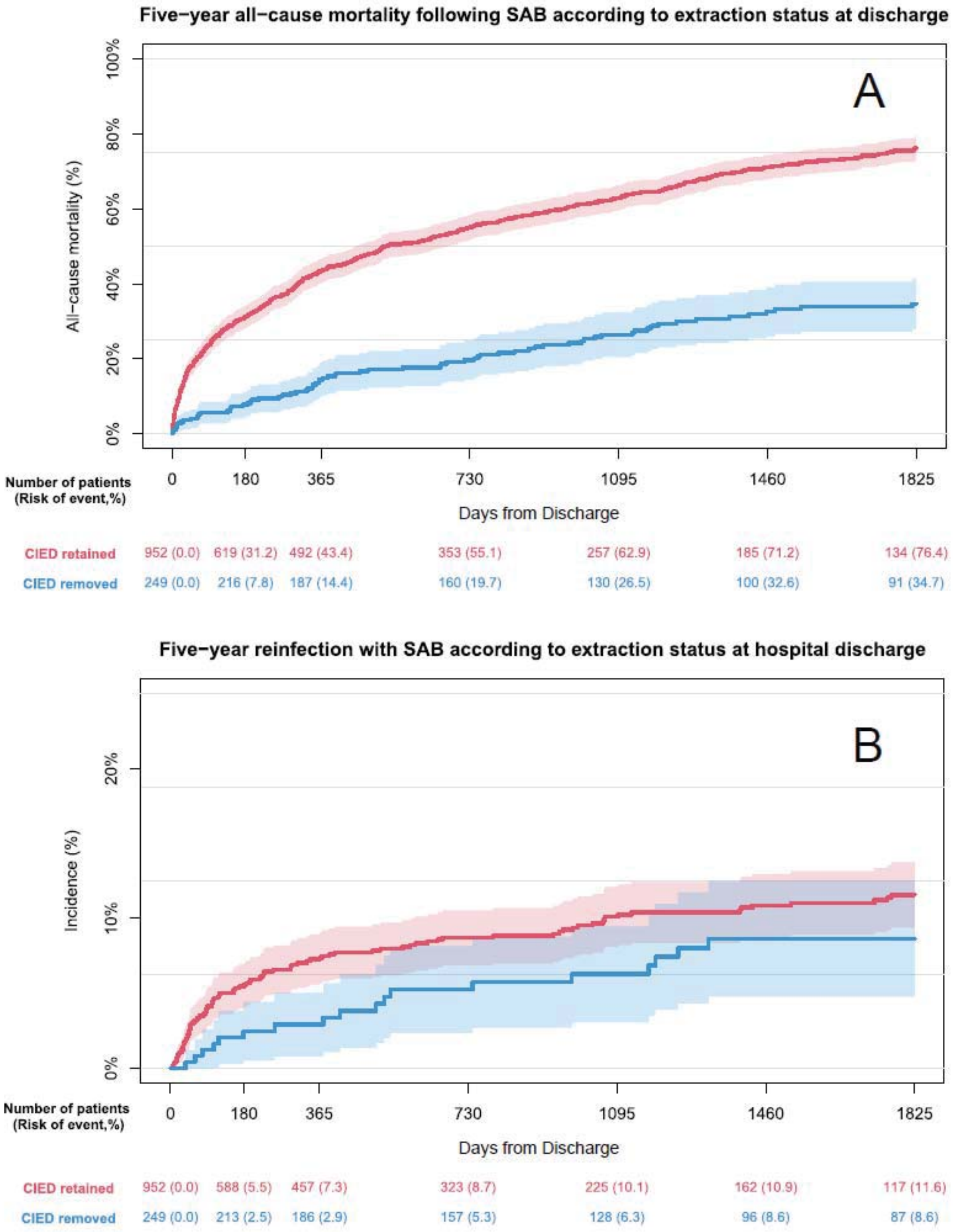

Figure S3 A+B. Landmark analysis at the time of hospital discharge of 5-year absolute risk of all-cause mortality (A) and 180-days cumulative incidence of reinfections (B) following SAB in patients with CIED stratified according to CIED removal status at discharge. Abbreviations: CIED: cardiac implantable electronic device, SAB; *Staphylococcus aureus* bacteremia
